# Towards a sterile insect technique field release of *Anopheles arabiensis *mosquitoes in Sudan: Irradiation, transportation, and field cage experimentation

**DOI:** 10.1186/1475-2875-7-65

**Published:** 2008-04-25

**Authors:** Michelle EH Helinski, Mo'awia M Hassan, Waleed M El-Motasim, Colin A Malcolm, Bart GJ Knols, Badria El-Sayed

**Affiliations:** 1 International Atomic Energy Agency (IAEA), Agency's Laboratories Seibersdorf, A-2444 Seibersdorf, Austria; 2 Laboratory of Entomology, Wageningen University and Research Centre, P.O. Box 8031, 6700 EH Wageningen, The Netherlands; 3 Epidemiology Department, Tropical Medicine Research Institute, P.O. Box 1304, Khartoum, Sudan; 4 Medical Entomology Department, National Health Laboratories, Federal Ministry of Health, P. O. Box 287, Khartoum, Sudan; 5 School of Biological and Chemical Sciences, Queen Mary, University of London, Mile End Road, London E1 4NS, UK

## Abstract

**Background:**

The work described in this article forms part of a study to suppress a population of the malaria vector *Anopheles arabiensis *in Northern State, Sudan, with the Sterile Insect Technique. No data have previously been collected on the irradiation and transportation of anopheline mosquitoes in Africa, and the first series of attempts to do this in Sudan are reported here. In addition, experiments in a large field cage under near-natural conditions are described.

**Methods:**

Mosquitoes were irradiated in Khartoum and transported as adults by air to the field site earmarked for future releases (400 km from the laboratory). The field cage was prepared for experiments by creating resting sites with favourable conditions. The mating and survival of (irradiated) laboratory males and field-collected males was studied in the field cage, and two small-scale competition experiments were performed.

**Results:**

Minor problems were experienced with the irradiation of insects, mostly associated with the absence of a rearing facility in close proximity to the irradiation source. The small-scale transportation of adult mosquitoes to the release site resulted in minimal mortality (< 6%). Experiments in the field cage showed that mating occurred in high frequencies (i.e. an average of 60% insemination of females after one or two nights of mating), and laboratory reared males (i.e. sixty generations) were able to inseminate wild females at rates comparable to wild males. Based on wing length data, there was no size preference of males for mates. Survival of mosquitoes from the cage, based on recapture after mating, was satisfactory and approximately 60% of the insects were recaptured after one night. Only limited information on male competitiveness was obtained due to problems associated with individual egg laying of small numbers of wild females.

**Conclusion:**

It is concluded that although conditions are challenging, there are no major obstacles associated with the small-scale irradiation and transportation of insects in the current setting. The field cage is suitable for experiments and studies to test the competitiveness of irradiated males can be pursued. The scaling up of procedures to accommodate much larger numbers of insects needed for a release is the next challenge and recommendations to further implementation of this genetic control strategy are presented.

## Background

Application of the Sterile Insect Technique (SIT) entails the mass production, sterilization, and subsequent release of sterile male insects into a target population in an area-wide, and usually integrated, pest management strategy. The released males inseminate wild females with sterile sperm. The females subsequently fail to produce viable offspring leading to an overall size reduction of the target population. Over the years, SIT has proven to be a safe, effective and environmentally sound method to suppress, eliminate or contain particular insect pest populations [1]. The International Atomic Energy Agency (IAEA) has a long history of supporting SIT programmes against major insect pests, including fruit flies, tsetse flies and codling moths. In 2004, the IAEA initiated a five-year study to develop technologies for controlling malaria mosquitoes with the SIT [2,3].

The use of the SIT as a genetic control strategy for mosquitoes is not new. The majority of studies on genetic control of mosquitoes were conducted from 1950–1980. The induction of dominant lethality by radiation or sterilizing chemicals was perhaps the most researched area [4], but other forms of genetic control, e.g. translocations or other chromosomal rearrangements, were also undertaken. Benedict and Robinson [5] provide a review of the release programmes performed. The largest SIT release programme against an *Anopheles *vector (*Anopheles albimanus*) was executed in El Salvador in the 1970s [6]. Over a 5-month period, 4.3 million mosquito pupae were mass-produced, sterilized, and released around Lake Apastepeque. Analysis of *An. albimanus *population data [5] from the release and a nearby control area demonstrates how successful the sterile males were in preventing a normal seasonal rise in vector density [7]. A subsequent, more extensive trial, located on the Pacific coast of El Salvador, took place from 1977–79 [8,9] when up to 0.5 million sterile males or 1.25 million sterile male pupae were released daily. Complete control was not achieved due to the immigration of females from surrounding areas, despite the introduction of a barrier zone [9].

**Figure 1:**
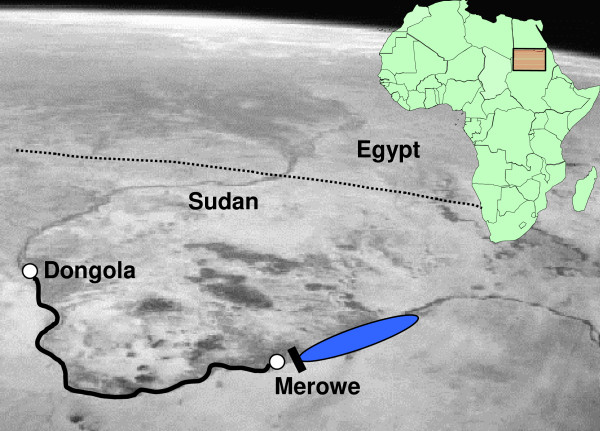
**Satellite image of the project area along the Nile, situated between Dongola and Merowe, in Northern State, Sudan.** The position of the Merowe dam and the reservoir lake are shown.

The Tropical Medicine Research Institute (TMRI) in Khartoum is leading a Republic of Sudan project to explore the possibility of using SIT as part of an integrated area-wide approach to control *Anopheles arabiensis *in the north-west of the country. The IAEA in collaboration with many partners is involved in the development and evaluation of the necessary components needed for an area-wide integrated approach to vector control of African malaria vectors using the SIT. Some of the experimental work is performed at the Agency's laboratories in Seibersdorf, Austria, and a pilot project area is under development in Sudan. The field site of the pilot project is situated in Northern State, where pockets of breeding sites of the malaria vector *An. arabiensis *occur on the banks of the Nile in an area otherwise surrounded by irrigated land and desert. The area stretches from Dongola in the north to Merowe in the south and is about 350 km long following the Nile (Figure 1). Upriver from Merowe, a dam is near to completion that will create a reservoir lake of approximately 200 km in length. *Anopheles arabiensis *is very rare along this stretch of the Nile and the lake will eliminate it altogether. The lake will be surrounded by rocky desert unsuitable for human habitation and so it should form a barrier to prevent migration of mosquitoes downriver. To the north of Dongola, conditions become far less favourable for *An. arabiensis*, due to the rocky terrain, sparse human population and colder climate. In addition, the Gambiae Control Project jointly run by the Egyptian and Sudanese Ministries of Health has been operating since 1970 along the Nile from Aswan in Egypt to Abu Fatma in Sudan. This programme has maintained and extended an *An. arabiensis *free zone that now reaches Abri [10]. All mosquito breeding sites between Merowe and Dongola are found within 5 km of the river banks. Breeding sites close to the river are primarily natural breeding sites associated with the seasonal flooding of the river, while the majority of breeding sites inland is man-made and associated with agricultural practices. The human population in Northern State is around 600,000 and heavily concentrated along the Nile. Temperatures in the area fluctuate greatly and are between 7–47°C [10], and relative humidity ranges from five to 70%. *An. arabiensis *is the only malaria vector present [10].

The rearing and sexual sterilization by gamma radiation occurs in Khartoum, situated approximately 400 km south of Merowe. The field site and Khartoum are connected by air with two to three commercial 1 hr flights a week (i.e. to Dongola), or 6 hours by road. Irradiation studies had been undertaken at the IAEA [11] but to date, no irradiation of anophelines had been performed in Sudan, and no irradiated males had been transported from Khartoum to the field site. In this study, initial experiences on the irradiation and subsequent transportation of adult mosquitoes to the field site by air are reported.

A field cage was constructed in Dongola to perform a variety of experiments under near-natural conditions [12], with emphasis on the survival and mating competitiveness of irradiated males against wild mosquitoes collected from the surrounding area. These experiments are vital in determining the true mating competitiveness of irradiated males. Only limited data on survival had previously been collected of insects placed in small rearing cages in the field cage. These indicated that survival was poor, and conditions in the field cage had to be improved to create more favourable conditions for mosquitoes. The preparation of the field cage for experiments is described and data on mating and survival of wild and laboratory-reared mosquitoes is presented. Of key importance was to assess if the laboratory-adapted males would withstand transportation to the field site and survive and mate under field conditions. In addition, two small-scale competition experiments were performed to investigate the competitiveness of irradiated males.

## Methods

### Mosquitoes

The laboratory strain used for all experiments is the Dongola strain of *An. arabiensis*. The strain was collected from breeding sites close to the town of Dongola and taken into culture in 2004. It was maintained both in the insectary in Seibersdorf (T: 27 ± 1°C, RH: 82 ± 2%) and in Khartoum for sixty generations (T: 26 ± 2°C, RH: 60 ± 10%) for use in the irradiation, transportation and field cage experiments. All wild mosquitoes used in the field cage experiments were collected from breeding sites in the vicinity of the field cage, either as larvae or pupae. They emerged in the Dongola insectary (i.e. situated in a building next to the field cage) and were held in standard rearing cages (30 × 30 × 30 cm) until release into the field cage. All mosquitoes were sexed < 18 hrs after emergence to ensure virginity. Adult mosquitoes were maintained in the insectary on sugar water (i.e. 10% sucrose solution).

### Irradiation and transport source

Insects were irradiated in a Cobalt-60 Gammacell (Nordion 220) following procedures described in Helinski *et al *[11]. The source was situated in Soba, approximately 45 min by car from the rearing facility in downtown Khartoum; thus insects had to be transported to and from the source. Insects were irradiated with a partially-sterilizing dose of 70 Gray (Gy) [13], and two separate irradiation sessions with different batches of insects were performed. Approximately 500 males were irradiated in each session. A dosimetry system was used to verify the dose received by the batch [11,13]. Males were irradiated in the pupal or adult stage, and pupae were sexed before irradiation. Pupal sexing was done manually by looking at the terminalia under a stereoscope; adult sexing was also done manually by visual determination. Pupae were transported to the source in a small holding container with a screw top to prevent spilling. Adults were transported in a standard rearing cage, placed in a stryrofoam box and covered with moist towels to avoid overheating of the mosquitoes.

### Transport to the Khartoum-field site

Un-irradiated males and two batches of irradiated males were taken by air as adults in separate trips to the field station in Dongola (Table 1). For transportation, adults were placed fifty at a time in standard paper drinking cups covered with cotton mesh. Sugar solution was provided in cotton wool secured on top of the cup. The cups were then fixed in place in a styrofoam box and covered with moist towels. Upon arrival in Dongola the boxes with mosquitoes were transported to the local insectary by car.

**Table 1:** Mortality of adult males during transport from Khartoum to Dongola by air.

Experiment	# Adult males transported	Mortality (%)
1	259	0
2	300	4
3	600	6

In experiment 1 and 2 irradiated males were used, in experiment 3 un-irradiated males.

### Field cage experiments

#### Preparation of field cage

**Figure 2:**
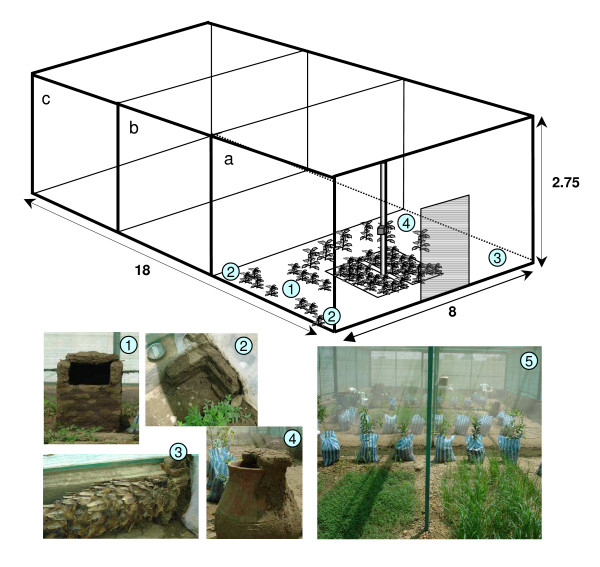
**Schematics of the field cage in Dongola.** The cage was divided in three equal sections (a-c); only for section a the various items are displayed but other sections were identical; Pictures 1–4 indicate the resting sites: 1) *kuseba*, 2) corner site, 3) tree trunk, 4) *zir*; position in field cage indicated by numbers. Picture 5 shows plant beds seeded with local crops and plants in sacks.

The field cage (18 × 8 × 2.75 m) used in the experiments consisted of a metal structure fixed with thick green shade netting that allowed for air and light exchange to simulate ambient conditions. The field cage was divided into three identical sections (6 × 8 m) to allow replicate experiments (Figure 2). Sections were made by gluing netting material to the metal structure, and clay and bricks were used to fix the netting at the bottom. Each section was accessible by an outside door. A datalogger, recording ambient temperature and relative humidity, was situated in each section at 1 m height, and recordings were made for two days (Table 2). Each section was equipped with resting sites consisting of a *kuseba *(i.e. a brick structure covered with clay, with a small opening where mosquitoes could enter), corner sites (i.e. brick/clay structures in the corners of the section), a tree trunk, and a *zir *(i.e. local clay pot closed partly with bricks; Figure 2). The *kuseba *and the *zir *were filled with a shallow amount of water to increase humidity. To further increase humidity and shelter, various types of vegetation were used. These consisted of two large plant beds in the middle of each section seeded with local crops such as bean and maize (Figure 2), and some smaller plant beds on the east side. Additionally, plants rooted in soil-containing sacks were distributed across the area (Figure 2).

**Table 2:** Climatic conditions in various sites in the field cage.

Day	4–5^th ^April 2007		
Site	*kuseba*	corner	ambient
Mean T (°C)	21.9 ± 3.1	24.8 ± 4.7	29.7 ± 6.8
Tmax	27.5	34.4	41.1
Tmin	17.5	19.4	19.0
Mean RH (%)	>95*	62.8 ± 10.7	16.3 ± 7.3
RHmax	100*	89	37.6
RHmin	57.6	35.9	6.5

Conditions were measured for two days in two resting sites in Section B, i.e. the *kuseba *and one of the corner sites in the left side of the field cage; ambient conditions are logged by the permanent logger in Section B at 1 m height. Mean (± s.d.), maximum, and minimum values are given. *logger got too wet to deliver precise readings.

#### Experimental procedures

Before introduction of mosquitoes, plant beds were flooded, and this was done every morning when experiments were run for more than one night. Two sugar feeders with a sucrose solution and a drop of honey were placed in each section; one in the *kuseba *and one close to a corner site, and refreshed every other day. Mating experiments lasted for one or two nights. In the latter case, mosquitoes were introduced in the early evening, in the former in the early afternoon. After mating, males and females were recaptured from the field cage by searching the resting sites and the whole section during the day. Mostly, mosquitoes were concentrated in a few places and these were checked carefully for approximately two hours. For the competition experiments, after the day collections, human landing catches in the early evening (i.e. from 8–10 pm) were performed.

Recaptured males were counted and a wing was clipped for size determination [14,15]. Recaptured females were either dissected to determine insemination status by the examination of the spermathecae for sperm, and a wing was collected, or kept for blood feeding and egg laying (i.e. in the competition experiments). With the exception of the first competition experiment wings from females were classified as coming from inseminated or un-inseminated females to determine a possible size preference of males. A digital image of the wing was taken (CC-12 camera, Soft Imaging System, Germany, mounted on a stereo microscope). Wing length was measured between the alula notch and the wing tip, excluding scales; measurements were performed with AnalySIS FIVE software (Soft Imaging System, Germany). In some experiments the males or females were dusted with fluorescent powder prior to release. Mosquitoes were placed in a plastic cup that was sealed at the top, and powder was applied with a syringe. Dusting was done to distinguish groups of mosquitoes after recapture and to identify potential survivors from the previous experiment (i.e. when experiments were performed in close succession).

#### Control experiments

Control experiments were performed to test the mating performance and survival of wild or laboratory reared males (i.e. transported from the insectary in Khartoum by air) when confined with wild virgin females. For each group of males, three replicates were performed with different batches of mosquitoes (with the exception of experiment 3 where the same batch of laboratory males was used for the two replicates). Age of the males and females used in the experiments was between two and five days, and mosquitoes were introduced at either a 1:1 ratio, or a higher ratio of females was used (Table 3). In the first experiment mating was allowed for two nights; in the second and third experiment mating lasted for one night.

**Table 3:** Data from control experiments where wild or laboratory reared males were introduced with wild females.

Experiment	# ♂ lab (age in d)	# ♂ wild (age in d)	# ♀ wild (age in d)	Ratio ♂: ♀	Recapture (%)		Insemination	
					♂	♀	N	%
1 wild males Section A	-	275 (2–3)	254 (2–3)	1:1	51	28	55	96

2 wild males Section A	-	60 (3)	100 (3)	1: 1.66	25	55	42	81
2 lab males Section B	60 (5)	-	100 (3)	1: 1.66	46	55	39	72

3 lab males Section A	60 (4)	-	100 (3–4)	1: 1.66	67	60	56	46
3 wild males Section B	-	60 (4)	100 (3–4)	1: 1.66	72	50	40	43
3 lab males Section C	60 (4)	-	100 (3–4)	1: 1.66	97	57	44	18

The number and age (i.e. between brackets) of the mosquitoes introduced is given, as well as the ratio of males and females used. Recapture rates of males and females from the field cage after mating, as well as insemination rate and number of females dissected (N) are presented.

#### Competition experiments

Mosquito irradiation for these experiments was performed in Khartoum and adults were transported by air (Table 1). Because the number of irradiated mosquitoes was low, only two experiments with different batches of insects were performed. Age of the mosquitoes at the start of the experiment was between two to four days (Table 4) and mating lasted for two nights. Irradiated males competed against wild males for wild virgin females. For the first experiment, males irradiated with 70 Gy as pupae or adults were combined, and introduced at a ratio of ~2 irradiated males versus one wild male. In the second experiment, all males were irradiated as pupae with 60 Gy, and introduced at a 1:1 ratio with wild males (Table 4). In experiment two, wild and irradiated males were dusted with different colours to distinguish them after recapture. The recovered females were blood fed on a human arm, and females were placed in individual vials for egg laying (experiment 1) or egg laying occurred *en masse *(experiment 2).

**Table 4:** Competition experiments in the field cage.

Experiment 1	# ♂* (age in d)	# ♂ (age in d)	# ♀ (age in d)	# ♀ lab (age in d)	Ratio ♂*:♂:♀	# ♀ recaptured		Inseminated (N)
						am	pm	
Section A first batch ♀	256 (2)	110 (2–3)	170 (2–3)		~2:1:0.6	19	2	
Recapture (%)	n/d		12					100 (3)

Section A second batch ♀				175	n/n	43	3	
Recapture (%)	5			26				63 (30)

Experiment 2 section B	223 (4)	223 (2–4)	223 (2–4)		1:1:1	51	2	
Recapture (%)	34	38	24					72 (18)

The number and age (i.e. in days between brackets) of the mosquitoes introduced is given, as well as the ratio of irradiated males (♂*) versus wild males (♂) versus wild females (♀; for the 2^nd ^batch of females in exp. 1 this ratio was not known). In experiment 1, first a batch of wild females was introduced, and secondly laboratory females. For females the number recaptured by daytime checking (am) and human landing catches in the evening (pm) is given. The proportion of recaptured males and females after mating, as well as insemination rate (%) and number of females dissected (N) after recapture are given. n/d: not determined, n/n: not known.

### Statistical analysis

General Linear Models (GLMs) or individual t-tests were used to compare the results from different treatments, and means were separated using Tukey's Honestly Significantly Different (HSD) tests. All tests were two-sided and performed using the SPSS software version 14 (SPSS Inc., Chicago, USA).

## Results

### Irradiation and transportation

The first pupal irradiation session resulted in some pupal mortality after irradiation and this was attributed to problems associated with the container used (i.e. some spilling had occurred during transport). In the second session, the container used for holding was properly sealed and pupal mortality was low (i.e. < 5%). The adults used in the first irradiation session had barely emerged upon transportation to the source, and a mortality of approximately 50% upon arrival and after cooling and irradiation was observed. For the second session, older adults were irradiated and transportation and cooling went well, however spills of the sugar source on the return journey caused substantial mortality. Dosimetry confirmed that the dose received during the first irradiation session was close to 70 Gy, however in the 2^nd ^session the dose received by the batch was slightly lower (i.e. 60 Gy).

Transportation of adults to the field site by air was successful. For the first batch of males no mortality was observed upon arrival and insects spend approximately five hours in the cups (Table 1). In the second and third batch, mortality was slightly higher because males spent more time in the cups (i.e. seven hours), and in the last replicate a cardboard box was used instead of stryrofoam, but overall mortality remained below 6% (Table 1).

### Field cage experiments

**Figure 3:**
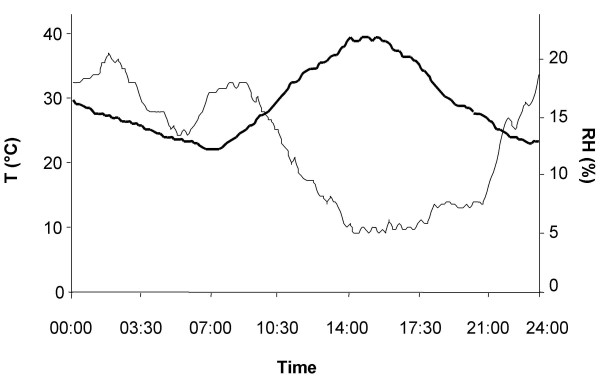
**Typical temperature (bold line) and relative humidity patterns in the field cage in April logged at 1 m height in section C over 24 hrs.** The increase in humidity in the morning is associated with the flooding of the plant beds.

Data from the three loggers indicated that the three sections were identical in ambient conditions. The fluctuation of temperature and relative humidity over 24 hrs is shown in Figure 3. Overall, humidity was low and dropped during the day when temperatures peaked, and temperatures fluctuated by 20°C over 24 hrs. The east side of the field cage was cooler and the majority of resting mosquitoes sampled during the day were observed in this part for all sections. Preferred resting sites were the *kuseba*, the corner sites, and the lower parts of the vegetation close to these sites. Data loggers placed in these sites showed that temperature was lower, and humidity higher compared to ambient conditions (Table 2). All mosquitoes were recaptured close to the ground, i.e. at less than 0.5 m height. The human landing catches performed in the competition experiments yielded less than five females (Table 4).

#### Control experiments

When wild males and females were introduced at a 1:1 ratio, insemination of the females was 96% after 48 hrs (Table 3). Even though the ratio of females was increased and mating reduced to one night only in the second experiment, wild males inseminated 81% of the females, and the laboratory males performed only slightly less with 72% of the females inseminated. In the third experiment, conditions were similar as in experiment two; however insemination rate was lower. Wild and laboratory males in sections A and B of the field cage inseminated 43% and 46% of the females, respectively (Table 3), and insemination of females mated to laboratory males in section C was only 18%. The reasons for this lower and variable insemination are not clear. No significant differences were observed in mean insemination for laboratory and wild males when combining the three replicates (t(4) = 1.26, p > 0.05).

After two nights of mating in experiment one, 28% of the females and 51% of the wild males were recaptured (Table 3). More mosquitoes were recaptured the following day (i.e. around 20), thus some mosquitoes escaped collection, and this was observed in other experiments as well. In experiments two and three, mating was restricted to one night and the recapture rate of females was between 50–60%. Recapture of wild males was highly variable and ranged from 25–72%, and the same applied to laboratory males with rates ranging from 46–97% (Table 3).

#### Competition experiments

After two nights of mating in experiment one, only a small fraction of the wild females introduced was recaptured (Table 4). Because a substantial number of males were seen alive in the cage, a second batch of females (i.e. dusted laboratory) was introduced. The following day around 26% of dusted females were recaptured. Recapture of the males after 84 hrs was only 5%. Blood feeding of the recaptured females was difficult; in the first batch, none of the wild females fed blood after several opportunities to do so. Few females from the second batch fed (N = 10), and only three egg batches were obtained. Hatch data showed that one batch had a hatch rate of 90% and was thus fathered by a wild male, while the other two batches had 0% hatch and were likely to be the result of a mating with an irradiated male. Unfed alive females were dissected to determine insemination status. From the first batch of wild females, three were dissected for insemination and all were inseminated; in the second batch 63% of females (N = 30) were inseminated (Table 4).

In the second experiment, an equal proportion of irradiated and wild males was recaptured after mating. Of the recaptured females 56% fed (N = 30). Three days after the blood meal, the alive fed females were flown back to Khartoum for *en masse *egg laying (N = 18), however, only 102 eggs were laid with a hatch rate of 11%. The unfed females (N = 18) were dissected for insemination and 72% of these were inseminated (Table 4).

#### Wing length data

For all experiments (i.e. control and competition) combined, the wild males were smaller than laboratory reared males (t(220) = -6.62, p < 0.01; Table 5). However, no size-differences were observed between wild and laboratory males in control experiment two (t(6.35) = 0.24, p > 0.05) or three (t(62) = 0.37, p > 0.05; Table 5). Although no samples were available of irradiated males in competition experiment one, the males recaptured after mating probably were irradiated laboratory males as their wing size was significantly larger (i.e. 2.70 ± 0.05 mm, N = 16; t(21) = -2.60, p < 0.05) compared to the wild males sampled before introduction.

**Table 5:** Mean (± s.e.m.) wing lengths (mm) of males from competition and control experiments.

Experiment	Wild		Laboratory	
			irradiated†/un-irradiated	
	N	size	N	Size
Competition				
1	8	2.47 ± 0.07		n/d
2	47	2.67 ± 0.02b	39	2.88 ± 0.02a†

Control				
1	41	2.60 ± 0.02		n/a
2	7	2.75 ± 0.09a	16	2.73 ± 0.02a
3	25	2.74 ± 0.03a	14	2.77 ± 0.04a section A
			25	2.70 ± 0.04a section C

Overall	128	2.65 ± 0.01a	94	2.78 ± 0.02b

N is the number of wings measured. Mean values for wild and laboratory (i.e. irradiated and un-irradiated) males are given. Means without letters in common are significantly different at p < 0.05 for each row. n/d: not determined, n/a: not applicable.

The wild females from competition experiment one (i.e. 2.62 ± 0.03 mm, N = 17) were strikingly smaller than the second batch of laboratory females introduced (i.e. 2.95 ± 0.05 mm, N = 21; t(36) = -5.46, p < 0.01). The wild females from all other experiments were larger and similar in size compared to the laboratory females from competition experiment one (t(204) = -1.41, p > 0.05). No size differences were observed between inseminated or un-inseminated females when data were combined (t(183) = 1.13, p > 0.05; Table 6). When data were analysed per experiment similar results were observed, except for control experiment three section A where the un-inseminated females were significantly larger than the inseminated females (t(23) = -3.83, p < 0.01; Table 6).

**Table 6:** Mean (± s.e.m.) wing lengths (mm) of wild females recaptured from competition and control experiments.

Experiment	Section	Females mean wing length (mm) ± s.e.m.			
		inseminated		un-inseminated	
		N	size	N	Size
Competition					
2	B	11	2.86 ± 0.05a	6	2.83 ± 0.08a

Control					
1	A	40	2.88 ± 0.02a	2	2.91 ± 0.01a
2	A	25	2.94 ± 0.02a	6	2.90 ± 0.07a
	B	22	2.98 ± 0.03a	9	2.95 ± 0.06a
3	A	13	2.81 ± 0.05a	12	3.03 ± 0.03b
	B	5	2.83 ± 0.10a	11	2.81 ± 0.08a
	C	6	2.84 ± 0.13a	17	2.73 ± 0.06a

Overall		122	2.90 ± 0.01a	63	2.86 ± 0.03a

N is the number of wings measured. Wings were grouped according to insemination status. Means without letters in common are significantly different at p < 0.05 for each row.

## Discussion

The first series of attempts to irradiate and transport anopheline mosquitoes from the laboratory to a remote field site in the context of a SIT study in Sudan are reported in this paper. Logistically, the project in Sudan is a challenging one with time consuming travel between the rearing facility and the irradiation source and considerable distances to the field site. The irradiation of insects performed in this paper was the first irradiation of *Anopheles *mosquitoes in Sudan or in Africa overall. Some difficulties were observed for pupal and adult stage irradiation, and these were associated with transportation and cooling of the insects, and a general lack of experience with performing the experiments under local conditions. The irradiation process was excluded as the likely cause of problems as similar experiments performed in a well-controlled laboratory environment showed no impact of pupal irradiation on emergence or of adult cooling on recovery [11].

The transportation of adults to the field site by air in relatively small numbers was successful. For future release programmes, much larger numbers of insects will have to be transported, and the next step would be to scale up transportation for these kinds of numbers. The distance of the field site from the irradiation source is likely to result in adult transportation even if pupal irradiation is performed, and devices that can transport adults or allow for adult emergence during transport should be explored. The transportation and ground release of adults was performed in the El Salvador release trials in the 1970s. A special "flat cage" was developed that could hold up to 2,000 adults and cages could easily be stacked for transport [16]. Mortality was acceptable; however the handling was intensive and caused considerable stress to the mosquitoes. Releases were difficult and, due to the weather conditions, adults had to be released after sunset [16]. It is known that cooling can be used to slow down pupal development (Helinski, unpublished data), however, it remains to be tested if after irradiation the development of the pupae can be slowed down successfully to allow for pupal releases. The release of pupae was also performed in El Salvador. Pupae were released in cups or pans and left to emerge in the field [8,16,17], and a cup could hold around 1,500 pupae. Cups were either put in floating containers that were released on water surfaces of breeding sites or on land when placed in release shelters [8].

Field cage experiments were successful in demonstrating mating and survival of released mosquitoes in the field cage. Data clearly indicated that mating occurred in the field cage, and a large proportion of females (i.e. on average 60%) was inseminated after only one or two nights of mating. Wild males appeared to perform slightly better than laboratory males but no significant differences were observed due to the variation observed between replicates. It is recommended that additional experiments are performed to understand the source of variation. The observation that laboratory males were capable of inseminating wild females suggests that no major behavioural differences due to the rearing process existed that impacted on mating and this is of great importance for an SIT project. To maintain this behaviour under mass-rearing conditions, introgression of wild material in the rearing colony, a strategy routinely performed in other mass-reared insects [18], could be adopted. There was no preference of males to mate females of a particular size, and wing lengths of inseminated and un-inseminated females were similar. This is in contrast to a laboratory study performed with *Anopheles gambiae s.s*., where males were observed to select larger females as mates [19], but in agreement with a study performed in the wild where the size distribution of mated *Anopheles funestus *females was similar to the distribution observed after emergence [20].

Recapture of mosquitoes from the field cage was virtually all done by sampling the resting sites during the day. Climate data showed that even when ambient conditions were harsh, with high temperatures and low humidity, micro-climates could be created where conditions were favourable for mosquitoes. Preferred resting sites were the brick/clay structures and the vegetation in the corners, and the *kuseba*, both in the east side of the field cage. Human landing catches only caught few females when performed during the early evening (i.e. 8–10 pm). It remains uncertain whether this was due to the fact that all females had been recaptured during daytime or that the period for collection was not suitable. In a study done in Ethiopia, the majority of *An. arabiensis *collected with human landing catch were caught after 10 pm [21], and similar observations were made in Kenya [22]. However, in another study in Eritrea peak activity of *An. arabiensis *was observed between 8–10 pm and 1–3 am [23]. The proportion of mosquitoes recaptured after 1 night of mating was larger than after two, with recapture rates for the females between 50–60%, and males between 25–97%, although the latter value could have been overestimated by some males still present from a previous experiment. Recapture rates of laboratory males were similar to those of wild males. A low recapture rate was observed in the first competition experiment, and wild males and females were strikingly smaller in size, suggesting that small insects suffered greater mortality in the field cage. Data on male survival in the field are scarce, but data from field-collected *An. gambiae s.l*. females suggested that larger females had a higher probability of survival compared to smaller ones [14,24]. The smaller size of the wild females in competition experiment one was attributed to the fact that these females had been collected from a different breeding site compared to the rest of the females. Only few dead mosquitoes were found in the field cage, notably in the breeding sites, thus it remained uncertain if recapture rates reflected true survival. In some experiments, the day following recapture more mosquitoes were found, however, their numbers were low and it was reasonable certain to assume that a large proportion of mosquitoes alive in the sections were recaptured. Experiments were performed in close succession due to time limitations, but it is advisable in future experiments to allow more time between experiments.

Unfortunately, the number of eggs obtained from the two competition experiments performed was too low to draw any meaningful conclusions on the competitiveness of irradiated males. This was due to the low number of females introduced and recaptured and problems associated with the feeding and egg laying of wild females in the laboratory. In the first experiment, two out of the three egg batches obtained were classified as resulting from an irradiated male, and the ratio of irradiated males in this experiment was twofold compared to wild males. In the second experiment, larger numbers of fed females were obtained but egg laying was low. Hatch rate of the eggs was only 11% suggesting that the 1–2 females that laid eggs were inseminated by an irradiated male. In future experiments, it is recommended that only one night of mating is used to increase recapture rates. Preferably, a strain is used for competition experiments in which mating can be detected by PCR or other molecular methods (e.g. for instance the use of a (transgenic) genetic sexing strain [25]), or alternatively stable isotopes can be used to label the semen [26]; thus excluding the need for egg batches to determine sterility. The survival of irradiated males was comparable to wild males in experiment two, and also in experiment one the wing length data suggested that most recovered males belonged to the irradiated males group. Thus it appeared that irradiation does not impact on survival in the first nights and this is important for future studies on competitiveness.

Insects in this study were separated to sex by manual determination of either pupae or adults. For a large-scale SIT programme, this method is obviously not feasible and automated methods of sex-separation are required. In the near future sex-separation will be performed using a genetic sexing strain. Genetic sexing strains (GSSs) have been developed for various insects including anophelines and they rely on the linkage of a dominant selectable marker to the male determining chromosome or locus. Linkage is accomplished by radiation-induced translocations followed by crossing and screening of the offspring. Resistance genes, e.g. temperature sensitive lethal genes and insecticide-resistance genes, have been used as selectable markers. A successful anopheline GSS was the MACHO strain of *An. albimanus *used in the second trial at the Pacific coast in El Salvador [9]. Several GSSs for *An. arabiensis *are under development at the IAEA and promising results were observed with some. These strains will be evaluated for their use in SIT programmes in the near future.

## Conclusion

It is concluded that the irradiation and transportation of insects to the field site as performed in this study is feasible. Some difficulties were experienced with the irradiation process (i.e. transportation and cooling) but these will be overcome in the near future when the new rearing facility is established in close proximity to the irradiation source. In the meantime, lessons learned from adult transportation to the field site can be applied to minimize mortality. Ultimately, the irradiation and transportation of much larger numbers of insects is needed for a release, and focus should lay on the development of such tools. The field cage experiments demonstrated that mating occurred in high numbers and recovery of released insects was satisfactory. Irradiated males survived and mated in the field cage and further experiments on the competitiveness of these insects should be pursued.

## Authors' contributions

MEHH and MMH performed the experiments and MEHH drafted the manuscript. WME–M assisted with the experiments. BGJK helped plan the experiments and the preparation of the field cage, and supervised the writing of the manuscript. CAM and BE–S organized the experimental work and secured funding. All authors read and approved the final manuscript.
